# Dynamically unpolarized single-photon source in diamond with intrinsic randomness

**DOI:** 10.1038/srep46722

**Published:** 2017-04-26

**Authors:** Naofumi Abe, Yasuyoshi Mitsumori, Mark Sadgrove, Keiichi Edamatsu

**Affiliations:** 1 Tohoku University, Research Institute of Electrical Communication, Sendai, 980-8577, Japan

## Abstract

Polarization is one of the fundamental properties of light, providing numerous applications in science and technology. While ‘dynamically unpolarized’ single-photon sources are demanded for various quantum applications, such sources have never been explored. Here we demonstrate dynamically unpolarized single-photon emission from a single [111]-oriented nitrogen- vacancy centre in diamond, in which the single-photon stream is unpolarized, exhibiting intrinsic randomness with vanishing polarization correlation between time adjacent photons. These properties not only allow true random number generation, but may also enable fundamental tests in quantum physics.

In classical optics, the polarization state of light is represented by Stokes parameters or the Poincaré sphere^1^. Pure (linear, circular and elliptical) polarization states correspond to the surface of the sphere while unpolarized light is at the center of the sphere, unbiased relative to any pure polarization state. In quantum optics, polarization of a single photon is expressed by a two-level system, i.e., a qubit^2^, and the polarization state is represented by a density matrix or a Stokes vector. The unpolarized state of a single photon corresponds to the completely mixed state of a qubit. This means that the unpolarized state of a single photon is a statistical mixture of any two orthogonal polarization bases. If we measure the polarization of a single photon in a certain basis, the measurement outcome will be either of the two orthogonal polarizations with even probability, regardless of the choice of basis. The unpolarized mixed state of a single photon is crucial to explore fundamental problems for mixed states such as testing error-disturbance relations of measurements^3^,^4^ and exploring the nature of mixed states themselves^5^,^6^, as well as to realize genuine random number generators^7^,^8^,^9^. For these applications, in addition to the statical statistics predicted by the density matrix, we must examine the dynamical statistics of the measurement outcomes. In particular, to ensure true randomness it is essential to show that the polarizations between photons are not correlated with each other. However, to our knowledge, such dynamically unpolarized single photon sources have not been explored to date.

Negatively charged nitrogen-vacancy (NV) centres have received broad attention as single-photon sources over the last few decades^10^,^11^,^12^,^13^. These properties have allowed NV centres to be used in applications ranging from single-photon sources for quantum cryptography^14^,^15^ to tests of the foundations of quantum physics^16^,^17^,^18^. Negatively charged NV centres consist of a substitutional nitrogen atom and an adjacent vacancy with an excess electron in diamond (Fig. 1(a)). When NV centres are non-resonantly excited from the ground state (^3^*A*) to the excited state (^3^*E*) by a 532 nm laser, the photoluminescence spectrum consists of the zero-phonon line at 637 nm and broad phonon sidebands (600–850 nm) and the lifetime of ^3^*E* is 11.6 ns^10^. The excited states ^3^*E* are composed of an orbital doublet of *E*_*x*_ which emits horizontally polarized photons and *E*_*y*_ which emits vertically polarized photons. Electric dipole transitions are allowed for dipoles associated with *E*_*x*_, *E*_*y*_ in the plane perpendicular to the NV symmetry axis and perpendicular to each other due to the C_3v_ symmetry of the structure of a NV centre^19^,^20^ (Fig. 1(a)). In [111]-oriented NV centres, which we used in this study, the orientation of these two dipoles are parallel to the diamond surface^21^,^22^,^23^,^24^,^25^.

The degeneracy of the orbital doublet ^3^*E* is lifted due to coupling to localized vibrational modes (the Jahn-Teller effect), thereby causing orbital mixing between *E*_*x*_ and *E*_*y*_ within the lifetime of ^3^*E*^13^,^24^,^26^,^27^,^28^. The polarization visibility of emitted photons from a [111]-oriented NV centre around 4 K is below 60% for both the zero-phonon line under non-resonant excitation^24^ and the phonon-sidebands under resonant excitation^23^. This is likely due to the static Jahn-Teller effect^13^. From around 4 K to 50 K, the polarization visibility decreases to 0% (i.e., unpolarized) exhibiting *T*^5^ dependence with thermal equilibration of the orbitals^24^. This *T*^5^ dependence is clear evidence of the dynamic Jahn-Teller effect. This temperature dependence implies that single photons emitted from a [111]-oriented NV centre are also unpolarized at room temperature under non-resonant excitation (Fig. 1(b)). Furthermore, as in thermal noise generators^29^, it is reasonably expected that truly random selection takes place when photons are emitted from either the *E*_*x*_ or *E*_*y*_ orbital, leading to dynamically unpolarized single photon emission without polarization correlation between time adjacent single photons. However, as mentioned above, polarization correlation between single photons, including those emitted from a [111]-oriented NV centre, has until now never been examined.

## Results and Discussion

### Static polarization measurements

**Figure 1:**
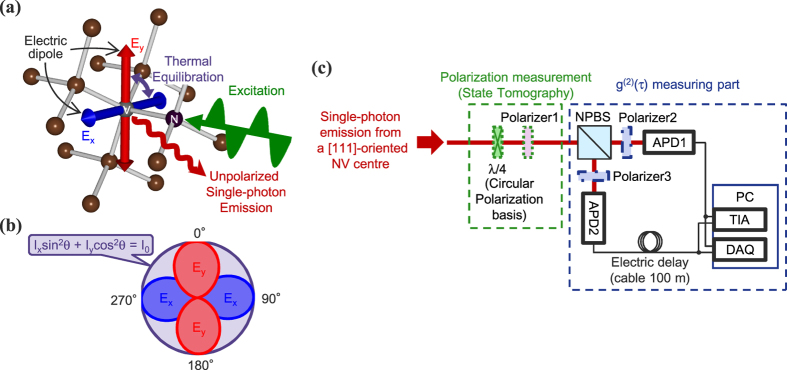
Structure of a [111]-oriented NV centre in diamond and experimental setup. (**a**) Structure of a NV centre in diamond and electric dipoles *E*_*x*_ and *E*_*y*_. (**b**) Schematic diagram depicting the unpolarized emission pattern (purple curve) caused by sum of emission from electric dipoles *E*_*x*_ (blue curve) and *E*_*y*_ (red curve) of a [111]-oriented NV centre. (**c**) The detection part of the experimental setup. For quantum state and process tomography, polarizer1 (purple rectangle) was inserted before a non-polarizing beamsplitter (NPBS). A *λ*/4 plate (green rectangle) was also inserted with its optic axis at 45° before the polarizer for measurement in the circular polarization basis. The setup for measurement of the second-order correlation function *g*^(2)^(*τ*) consisted of the NPBS, two avalanche photodiodes (APDs), a 100 m cable for electric delay and a time interval analyser (TIA). In other measurements, such as polarization angular dependences and tomography, pulses generated by APDs were counted by a data acquisition (DAQ) card. When we measured polarization correlations of the emitted single-photon stream, polarizer2, 3 (thick blue rectangles) were introduced between the NPBS and the APDs with HH, VV, HV and VH, where the first (second) character represents the polarization setting of polarizer2 (polarizer3).

**Figure 2:**
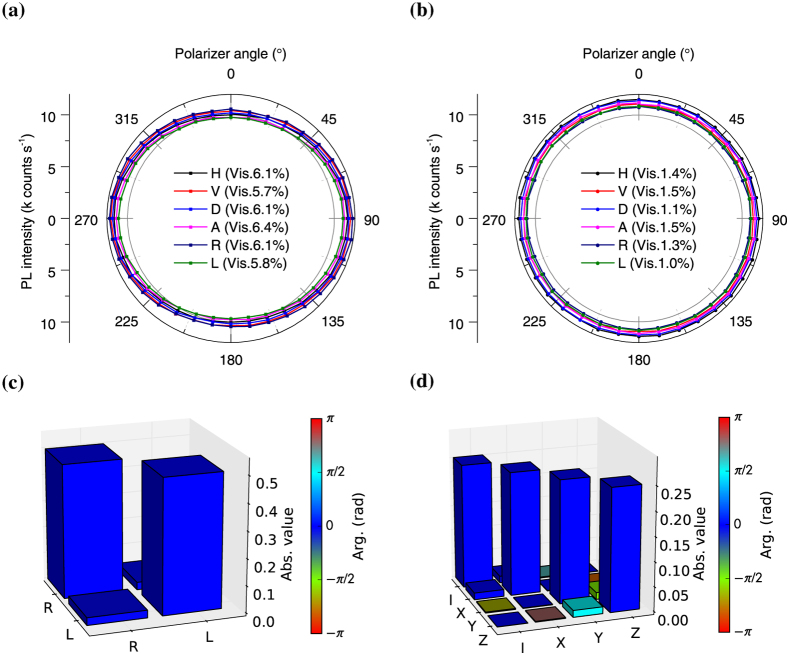
Polarization angular dependence and tomography. (**a**) Linear polarization angular dependence of fluorescence intensity from a [111]-oriented NV centre when excitation laser polarization was set to be horizontal (H), vertical (V), diagonal (D), anti-diagonal (A), right circular (R) and left circular (L). Vis. indicates the visibility of each curve. (**b**) Circular polarization angular dependence of fluorescence intensity from the NV centre when the laser polarization was set to be the same as (**a**). (**c**) The reconstructed density matrix of the polarization state of single photons emitted from the NV centre by quantum state tomography. The height of the bars indicates the absolute value of elements of the matrix and the colour represents the phase. (**d**) The *χ* matrix estimated by quantum process tomography for the excitation-emission process, when the the input (output) state of the process is assumed to be the polarization state of the 532 nm excitation laser (single photons emitted from the NV centre). The height and colours of the bars indicate the same as in (**c**).

First, we evaluated static polarization properties of emitted photons by measuring polarization angular dependencies, and applying quantum state tomography and quantum process tomography. The excitation by a 532 nm laser and the collection of the phonon sideband emission from single NV centres was carried out by a standard confocal microscopy method at room temperature (See Methods). The detection part of the experimental setup is shown in Fig. 1(c). We used type IIa (111) diamond synthesized by a high pressure high temperature (HPHT) method. In order to search for a [111]-oriented NV centre among four possible 〈111〉 orientations and investigate polarization states of the photons emitted from the NV centre, we measured detection polarization angular dependencies of the fluorescence intensity in both linear and circular polarization bases. In the linear polarization basis measurements, we measured the polarizer angle dependence of the fluorescence intensity by rotating polarizer1 (purple rectangle shown in Fig. 1(c)) for excitation laser polarizations set to horizontal (H), vertical (V), diagonal (D), anti-diagonal (A), right circular (R) and left circular (L). These data are shown in Fig. 2(a). We observed almost no angular dependence, with an average visibility of 6.0% for an excitation power of 1 mW (0.26*P*_sat_, where *P*_sat_ is the saturation excitation power). All measurements conducted in this paper were made for the same [111]-oriented NV centre. Furthermore, we confirmed similar unpolarized properties for several other [111]-oriented NV centres. In the circular polarization basis measurements, we measured the polarizer angle dependence of the fluorescence intensity by rotating polarizer1 followed by a *λ*/4 waveplate (green rectangle shown in Fig. 1(c)) at 45° for excitation laser polarizations of H, V, D, A, R and L as shown in Fig. 2(b). Again, we found only minimal angular dependence, with an average visibility of 1.3% for an excitation power of 1 mW.

#### Quantum state tomography

We reconstructed a density matrix *ρ*_exp_ for the polarization of the photons emitted from the NV centre from the data shown in Fig. 2(a,b) by a maximum likelihood quantum state tomography method^30^. The density matrix is shown in Fig. 2(c). The density matrix shows that the polarization state is nearly the completely mixed state which is given by


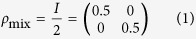


in either the R, L basis or the H, V basis, where *I* is the two-dimensional identity matrix. The Stokes vector calculated from the density matrix is (*S*_1_/*S*_0_, *S*_2_/*S*_0_, *S*_3_/*S*_0_) = (0.057, −0.003, −0.004). This vector is located near to the origin of the Poincaré sphere, indicating an almost completely unpolarized state. We also estimated the average fidelity 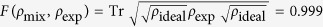. Therefore, the single photons emitted from the measured single [111]-oriented NV centre are statistically statically unpolarized.

#### Quantum process tomography

Next, in order to find a key quantum operator describing the excitation-emission process of the NV centre, we evaluated the *χ* matrix by quantum process tomography^31^,^32^ (See Methods). The input state of the process is assumed to be the polarization state of the 532 nm excitation laser and the output state of the process is assumed to be single photons emitted from the NV centre. The ideal *χ* matrix *χ*_ideal_ for the process, which outputs the completely mixed state *ρ*_mixed out_ for any input *ρ*_in_, is diagonal with all entries equal to 1/4 because of the identity relation *ρ*_mixed out_ = (*Iρ*_in_*I* + *Xρ*_in_*X* + *Yρ*_in_*Y* + *Zρ*_in_*Z*)/4 = *I*/2, where *X, Y* and *Z* are the Pauli matrices, which form the basis of the *χ* matrix. *χ*_exp_, which is the *χ* matrix estimated from the data shown in Fig. 2(a,b), is shown in Fig. 2(d). The result demonstrates that the process outputs a nearly completely mixed polarization state of emitted single photons for any input excitation polarization state. We also estimated the process fidelity^33^ 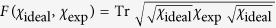 = 0.999.

### Dynamical polarization correlation measurements

**Figure 3:**
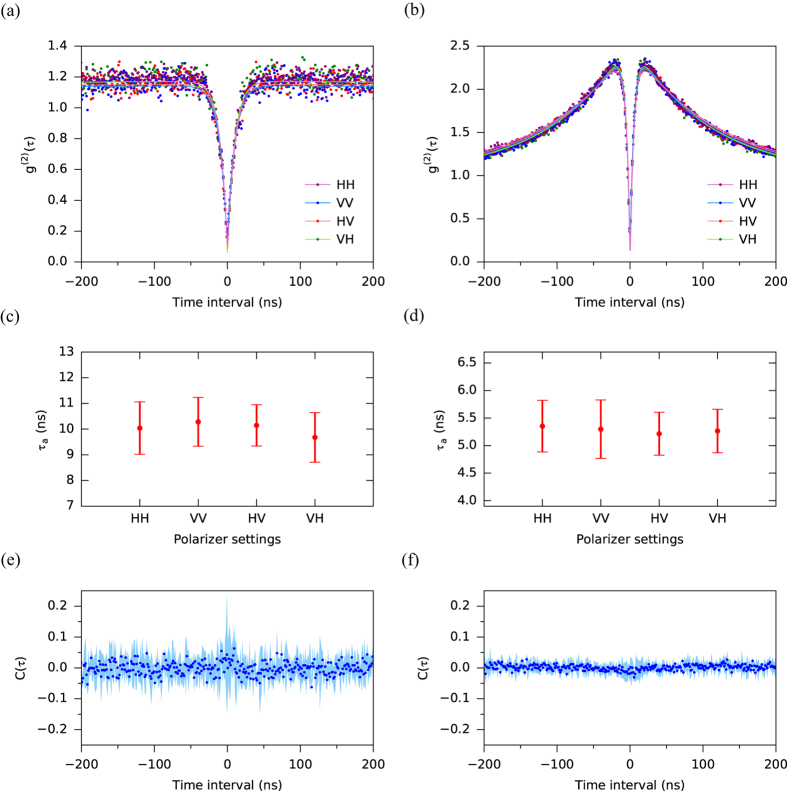
Polarization correlation measurements. (**a**) The modified second order correlation function 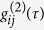. *ij* represents the polarizer settings HH, VV, HV and VH, where the first (second) character denotes the orientation of polarizer2 (polarizer3). The excitation power was set to be 0.20 mW (0.05*P*_sat_). The dots indicate the data and the lines indicate the fitting curves. (**b**) Same as (**a**) but for an excitation power of 8.8 mW (2.3*P*_sat_). (**c**) The decay times of anti-bunching estimated by fitting for the data shown in (**a**). The error bars indicate the standard error of the fitting parameter. Note that all points lie within the error bars of all the other points. This is convincing evidence of equivalence of the decay times. (**d**) Same as (**c**) but for the data shown in (**b**). (**e**) Polarization correlation function 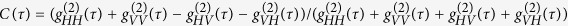 for the data shown in (a). The mean is 0.0008 and the standard deviation is 0.0206. (**f**) Same as (**e**) for the data shown in (**b**). The mean is 0.0022 and the standard deviation is 0.0109. The shaded areas in (**e**) and (**f**) indicate 90% confidence intervals.

Finally, in order to demonstrate the dynamical nature of the randomness of the emitted photon polarization, we measured the polarization correlation between time adjacent emitted single photons by measuring the modified second-order correlation function *g*^(2)^(*τ*) using the setup shown in Fig. 1(c). The basic setup was a standard Hanbury Brown-Twiss type setup in which the photon stream was divided by the insertion of a non-polarizing beamsplitter (NPBS), and detected at the outputs by avalanche photodiodes (APD1 and APD2). For the polarization correlation measurements, polarizer2, 3 (thick blue rectangles shown in Fig. 1(c)) were introduced between the NPBS and the APDs. We measured the modified second-order correlation function 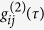 at weak excitation (0.20 mW = 0.05*P*_sat_), where *ij* denotes the polarizer settings HH, VV, HV and VH and the first (second) character denotes the orientation of polarizer2 (polarizer3). This means that if the polarizer settings are HV, APD1 detects only H polarization photons and APD2 detects only V polarization photons and *g*^(2)^(*τ*) is measured for these photons. If there is polarization correlation, for example time adjacent photons tend to have same H polarization, HH, the temporal profile of 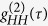 would give a narrower anti-bunching dip (i.e. a smaller decay time) than that of others, 
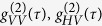
 and 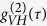. Thereby we can investigate dynamic polarization correlation using this method. The 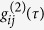 data we measured are shown in Fig. 3(a). The data show that the four 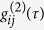 are identical to each other within experimental accuracy. The average of 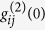 over the four polarizer settings was found to be 
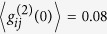
. Note that the measured value of *g*^(2)^(0) < 0.5 (anti-bunching) indicates strong evidence of single photons for all polarizer settings. These data indicate that time adjacent emitted single photons have essentially no polarization correlation. We also measured under strong excitation conditions (8.8 mW = 2.3*P*_sat_) as shown in Fig. 3(b). In addition to the anti-bunching dip with 
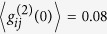
, bunching occurred around the anti-bunching dip due to optical cycling in the three-level system^10^. Again, we found that the four 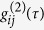 were identical to each other within experimental accuracy.

Usual *g*^(2)^(*τ*) data for emitted singe photons from a single NV centre are fitted by the following function which is derived by analysis of the three-level system,


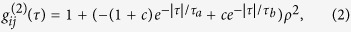


where *τ*_*a*_ is the decay time of anti-bunching, *τ*_*b*_ is the decay time of bunching, *c* is the coefficient of bunching and *ρ* is signal-to-background ratio^10^,^34^. For our cases, the 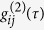 data were fitted by Eq. (2). Decay times *τ*_*a*_ of anti-bunching for the four polarizer settings (estimated by fitting for the 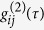 data shown in Fig. 3(a,b)) are shown in Fig. 3(c,d), respectively. These figures show that the decay times for the four polarizer settings are equal within the estimated standard error of the fitted values. These results are consistent with the expectation of uncorrelated decay times and we therefore conclude that the emitted single photons are dynamically unpolarized for weak and strong excitation cases.

Next, we evaluated the polarization correlation between same and different polarizations using a polarization correlation function *C(τ*) defined by


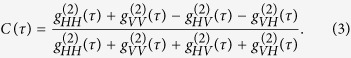


*C(τ*) is a quantitative measure of the dynamical polarization correlation. In particular, if measured photons have exactly the same (different) polarization, i.e., HH or VV (HV or VH), *C(τ*) is equal to +1 (−1) for any *τ*. On the other hand, if the measured photons have no polarization, *C(τ*) is equal to 0. *C(τ*) values for our data under weak and strong excitation condition are shown in Fig. 3(e,f), respectively. The data show that *C(τ*) is close to 0 for all *τ* that we measured; *C(τ*) averaged over all *τ* is 0.0008, 0.0022 and the standard deviation is 0.0206, 0.0109 for weak and strong excitation, respectively. Almost all of the 90% confidence intervals for *C(τ*) values (shaded area in Fig. 3(e,f)) include 0. Note that the slight peak and dip seen around *τ* = 0 in Fig. 3(e,f) respectively are due to fluctuations in the background which become prominent due to the vanishing correlation signal at *τ* = 0. We have therefore demonstrated essentially no polarization correlation between time adjacent emitted single photons for both weak and strong excitation conditions.

## Conclusion

We have presented the first demonstration of unpolarized single photons emitted from a [111]-oriented NV centre in diamond in both a statical and dynamical sense. The new ‘dynamically unpolarized’ property, i.e. the property of no polarization correlation between time adjacent single photons, constitutes a new criterion of randomness for unpolarized single photons which is useful for true random number generation^7^,^8^,^9^ as well as for tests of fundamental quantum mechanics pertaining to mixed states^3^,^4^,^5^,^6^.

## Methods

### Confocal microscopy

A continuous-wave 532 nm laser was used for non-resonant excitation of the NV centre. The laser was focused with a 1.4 numerical aperture (NA) oil immersion objective lens, which also collected the photons emitted from a single NV centre. Using band-pass filters, photons emitted from the NV centre ranging from 650 nm to 800 nm were measured. The measurements of the second-order correlation function 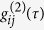 for the filtered photons were performed using a modified Hanbury Brown-Twiss type setup as explained in the main text.

### Quantum process tomography

Quantum process tomography is a method for estimating the *χ* matrix, which finds quantum operators describing a given quantum process^31^,^32^. The process 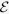 for the one qubit case, having input state *ρ*_in_ and output state *ρ*_out_ can be written as


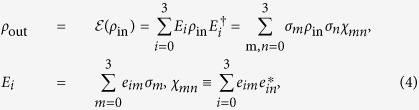


where *σ*_*m,n*_ ∈ {*I, X, Y, Z*}. Here, *I* is the two-dimensional identity matrix and *X, Y* and *Z* are the Pauli matrices, which form the basis of the *χ* matrix.

For our case, accurately speaking, the polarization state of this laser is a classical polarization rather than a quantum state. However the excitation-emission process is a linear process which means that a single-photon in the excitation laser is absorbed by a NV center and subsequently a single-photon is emitted from the NV center. In this case the polarization state of the absorbed single photon is identical to that of the excitation laser. This allows us to evaluate correlations arising from the process of excitation followed by emission of a single photon. We followed the concrete procedure of quantum process tomography given in ref. 32.

## Additional Information

**How to cite this article:** Abe, N. *et al*. Dynamically unpolarized single-photon source in diamond with intrinsic randomness. *Sci. Rep.* **7**, 46722; doi: 10.1038/srep46722 (2017).

**Publisher's note:** Springer Nature remains neutral with regard to jurisdictional claims in published maps and institutional affiliations.
